# 

**Published:** 1876-01

**Authors:** 


### Contents of January Number.

### ORIGINAL.

ART. I.—On the use of Warm and Hot Water in Surgery. By Frederick E. Hyde, M. D.,.......... 201

ART. II.—Medical Society of the County of Albany. Semi-Monthly Meeting, December 15th, 1875,.......... 215

ART. III.—Abstract of the Procedings of the Buffalo Medical Association, Dec. 7th, 1875,.......... 225

### MISCELLANEOUS.

Sudden Death after Uterine Injection,.......... 227
Internal Strangulation of the Intestine by Cicatrices,.......... 228
Epidemic Puerperal Fever,.......... 229
A Case of Inversion of the Uterus, with operation by White's Method,.... 232
Poisoning by Chloral,.......... 234

### EDITORIAL DEPARTMENT.

International Medical Congress,.......... 235
The Association of the Alumni and officers of the Medical Department of the University of Buffalo,.......... 237
Hospital Vacancy, etc.,.......... 238

BOOKS REVIEWED:

Lectures on Diseases of the Nervous System. By Jerome K. Baudny, M. D.,.......... 239
Clinical Lectures and Essays. By Sir James Paget, Bart.,...... 239

Books and Pamphlets Received,.......... 240

